# Validation and exploratory factor analysis of Urdu Version of Chronic Liver Disease Questionnaire

**DOI:** 10.12669/pjms.39.1.6602

**Published:** 2023

**Authors:** Sana Muhammad Hussain, Bader Faiyaz Zuberi, Tazeen Rasheed, Faiza Sadaqat Ali, Erum Majid

**Affiliations:** 1 Sana Muhammad Hussain, Department of Medicine/Gastroenterology, Dow Medical College, Dow University of Health Sciences, Karachi, Pakistan; 2 Bader Faiyaz Zuberi, Gastroenterologist & Hepatologist, Karachi, Pakistan; 3 Tazeen Rasheed, Department of Medicine/Gastroenterology, Dow Medical College, Dow University of Health Sciences, Karachi, Pakistan; 4 Faiza Sadaqat Ali, Associate Professor, Ward-9, Jinnah Postgraduate Medical Centre, Karachi, Pakistan; 5 Erum Majid, Associate Professor, Ward-9, Jinnah Postgraduate Medical Centre, Karachi, Pakistan

**Keywords:** Cirrhosis, Quality of Life, CLDQ, Urdu, Exploratory Factor Analysis

## Abstract

**Objective:**

To present and validate psychometric properties of Urdu version CLDQ, yet another objective was to do exploratory factorial analysis (EFA) of CLDQ Urdu version.

**Methods:**

This Cross-sectional Analytical Study was conducted at Dr. Ruth K.M. Pfau Civil Hospital Karachi during the period Nov. 15, 2021 to Jan. 30, 2022. CLDQ Urdu questionnaire was self-administered by the patients. The questionnaire consisted of 29 items and responses were recorded on 7-point Likert type scale. Reliability testing was done by Cronbach’s α, test value of >0.7 is taken as reliable. Exploratory factor analysis (EFA) was conducted with principal component analysis with varimax rotation. Adequacies for conduction of EFA depended on Kaiser-Meyer-Olkin (KMO) value of ≥0.5 and Bartlett’s Test of Sphericity (BTS) of ≤0.05. Mean CLDQ Urdu scores were also compared with Child Class using ANOVA and post-hoc analysis was done.

**Results:**

A total of 320 patients were selected after informed consent. All conditions for adequate EFA were met (Cronbach’s α =.949; KMO = .846; BTS ≤.001). Mean CLDQ Urdu score was 156.74 in male and 133.27 in female (*p*<.001). Child Class-A had best quality of life with score of 186.63 ±6.91 and Child Class-C had the worst with scores of 109.78 ±21.33. EFA resulted in reduction of domains to 4 (Muscular Symptoms, Emotional Symptoms, Abdominal Symptoms & Somnolence) & reduced the number of items from 29 to 11.

**Conclusion:**

Urdu CLDQ version is validated in our settings. EFA resulted in reductions in number of domains and items. CLDQ Urdu showed that quality of life decreases significantly with Child Class.

## INTRODUCTION

In the past twenty years, there has been an increasing apprehension that the traditional assessment of medical outcomes after medical interventions is inadequate. Therefore, health-related quality of life (HRQOL) gained significance as an outcome measure in clinical and epidemiological studies.^1^ Many generic and specific instruments are in vogue to measure HRQOL. Amid generic instruments, Medical Outcomes Study 36-Item Short Form Health Survey (SF-36),^2^ has been used widespread to postulate a global assessment of a given disease and allow its appraisals with general population and other diseases. Generic instruments are unable to evaluate disease-specific symptoms of liver disease, e.g., pruritus. They are less receptive to small, nevertheless clinically important, variations. Disease-specific instruments were developed to assess specific characteristics of a disease and provide an accurate instrument for clinical studies. Chronic Liver Disease Questionnaire (CLDQ) is disease-specific HRQOL instrument to evaluate different stages of liver diseases.^3^

Chronic liver disease (CLD) is a global health problem and is the 11^th^ leading cause of death per year. It badly affects the quality of life of CLD patients and accounts for one of the top 20 causes of global morbidity.^4^ Patients of chronic liver disease not only suffer from disease and its complications, therapeutic procedure related morbidity, side effects of drugs but also from emotional and psychological issues. Overall survival of CLD patients has increased due to advances in medical sciences. So along with survival, improvement in HRQOL and periodic assessment and monitoring of disease impact on patients health, physiological and socioeconomic status should also be prioritized.^5^

HRQOL includes physical, social, psychological, and functional aspect of disease and has influence on patient’s behavior and quality of life. It is categorized into generic and disease specific which provide health utilities and deals with single disease respectively.^6^ Chronic Liver Disease Questionnaire (CLDQ) is a disease specific instrument that was developed by Younossi et al in English language for the assessment of health related quality of life of chronic liver disease patients.^3^ It was later translated into many regional and international languages of world including Spanish, Italian, Greek, German, Swedish, Persian, Thai, Indian, Tamil, Japanese, Chinese, Bengali and Sri Lankan languages.^7^-^9^ Validity and reliability of CLDQ has been well established in many studies. The English version of this questionnaire has been validated in Pakistani population previously.^10^

Urdu is national language of Pakistan but Urdu version of CLDQ has not been developed yet for better understanding and assessment of HRQOL in Urdu speaking population. So, it is high time to formulate CLDQ in Urdu language for the assessment of health-related quality of life. Although CLDQ has been translated into many languages but only two, Spanish^11^ and Italian^12^ reported its factorial analysis which were much divergent with inconsistent results. While the Exploratory Factorial Analysis (EFA) of Spanish version was much near to original version of Younossi et al; the EFA of Italian version was much different from that of original.

There is imminent need for development of Urdu version of CLDQ and its EFA that would provide the assessment of HRQOL of chronic liver disease patients with items that are more lucid for our population.^5^ This study will help in developing an Urdu version of CLDQ questionnaire, its validation and EFA will yield important factors in Urdu version of questionnaire leading to better assessment of quality of life in local population. The objective if this study was to present and validate psychometric properties of Urdu version CLDQ, yet another objective was to do exploratory factorial analysis (EFA) of CLDQ Urdu version.

## METHODS

### Chronic Liver Disease:

It will be labelled if any one of the following criteria is present:

- Ultrasound showing deranged echo pattern
- Irregular liver margins
- Hepatomegaly
- Reduced liver size
- History of chronic liver disease for more than six months including chronic hepatitis
- Liver stiffness measured by transient elastography threshold of ≥9.5 kPa.^13^

### Chronic Liver Disease Questionnaire (CLDQ):

chronic liver disease questionnaire is a disease specific tool for the assessment of health-related quality of life (HRQL) in patients with chronic liver disease. CLDQ comprising of 29 items designated to measure six domains affecting quality of life of chronic liver disease patients. These six domains are fatigue, emotional function, abdominal symptoms, systemic symptoms, activity and worry.^3^

### Translation:

Urdu translation was done by three bilingual (English & Urdu) persons, whose native language was Urdu, using forward and backward translation technique. Translators were requested to maintain conceptual rather than linguistic equivalence. Any discrepancies in translations were resolved by mutual consensus of translators.

This Cross-sectional Analytical Study used Consecutive non-probability sampling technique and was conducted on patient admitted in medical wards and attending OPD of Dr. Ruth KM Pfau Civil Hospital Karachi during the period November 15, 2021 till March 31, 2022 after taking IRB approval, Ref No. (IRB-2253/DUHS/Approval/2021/576, dated Nov. 10, 2021).

### Sample Size:

For reliability analysis of questionnaire sample should be 10 times of the number of items as this questionnaire has 29 items, sample size was 290 patients were taken.

### Inclusion Criteria:

- All patients of Chronic Liver DiseaseAAAA
- 18-80 years of age1111
- Either genderEEEE

### Exclusion Criteria:

- Chronically ill patients due to any other chronic diseaseCCCC
- Patients with history of stroke, Chronic Kidney Disease, Chronic Obstructive Pulmonary Disease and Congestive Cardiac Failure.
- Patient with Encephalopathy (Metabolic and Hepatic)PPPP
- Patient with cognitive impairmentPPPP
- Malignancies including hepatocellular carcinoma.

All patient of chronic liver disease visiting outpatient’s department and admitted in medical wards of Dr Ruth KM Pfau Civil Hospital Karachi fulfilling the inclusion criteria were enrolled in the study during the period 15-11-2021 & 30-01-2022. Detail physical examination was carried out. Baseline characteristics and demographic data of each patient were collected on pre-designed proforma. CLDQ Urdu questionnaire was self-administered by the patients, one of the authors was available for resolution of any problem in filling out the questionnaire. The questionnaire consists of 29 items and response was recorded on seven point Likert type scale, where one was worst and seven was best. Blood sample of 10 ml was withdrawn by phlebotomist for estimation of Serum Albumin, INR, HBs Ag & HCV Ab. Child Class of all patients was calculated.

### Data analysis:

Demographics and etiology of liver disease etiology was described. Item analysis was based on calculated: means, ±SDs, and item discrimination. The proportion of patients who did not answer an item (missing) was used as a consideration of ‘‘acceptance.’’ Subscale level analysis was done. For each subscale, means and ±SDs were calculated. Reliability testing was done by internal consistency Cronbach’s α test, value of ≥0.70 was taken as acceptable. Exploratory factor analysis (principal component analysis with varimax rotation) was conducted. Only factors with an Eigenvalue ≥1 were interpreted. List wise exclusion of cases was done to decrease impact of missing data. Child Class of selected patients was calculated. Mean CLDQ Scores were compared with gender using Student’s t-test. Mean CLDQ Urdu scores were also compared with Child Class using ANOVA. Post-hoc analysis was also done to assess inter-category comparisons of CLDQ Urdu Scores. Significance level was ≤.05 and SPSS 26.0 was used for analysis.

## RESULTS

A total of 320 patients fulfilling inclusion/exclusion criteria were selected after informed consent, these included 201 (62.8%) males and 119 (37.2%) females. HCV Ab was positive in 236 (73.8%) and HBs Ag was positive in 84 (26.3%) patients. Break up of patients according to Child Class was Child Class A had 86 (26.9%) patients, Child Class B had 113 (35.3%) and Child Class C had 121 (37.8%) patients. (Table-I)

**Table-I:**
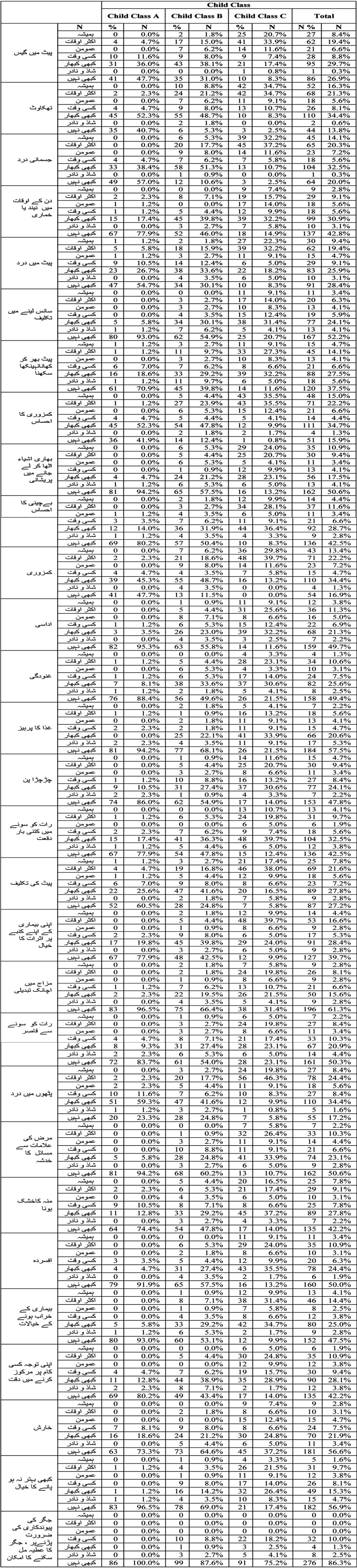
Details of CLDQ Urdu responses according to Child Class.

### Reliability Analysis:

The reliability analysis of all 29 items by Cronbach’s α gave highly significant value of .949. Two of 29 items (Itching & availability of liver for transplant) had communalities value of <0.5 and they were excluded, analysis repeated after exclusion resulted same Cronbach’s α value of .949 thus there was no effect on overall reliability.

### Factor Analysis:

Factor analysis was done after excluding two items that had poor communalities value of <0.5. The correlation matrix value was a positive value (.002). It must be positive value for proper factor analysis. Kaiser-Meyer-Olkin (KMO) and Bartlett’s Test of Sphericity (BTS) were done to see the fitness of data for factor analysis. KMO Measure of Sampling Adequacy was 0.846 which was more than the required value of 0.5. Bartlett’s Test of Sphericity showed p-value of <0.001 indicating adequacy for conducting the factor analysis. Factor analysis was done using Principal Component Extraction method for Correlation Matrix. Factors with Eigenvalue <1 was excluded. Varimax rotation was used for final factor reduction. KMO Measure of Sampling Adequacy was 0.846 which was more than the required value of 0.5. Bartlett’s Test of Sphericity showed p-value of <0.001 indicating adequacy for conducting the factor analysis.

**Fig.1:**
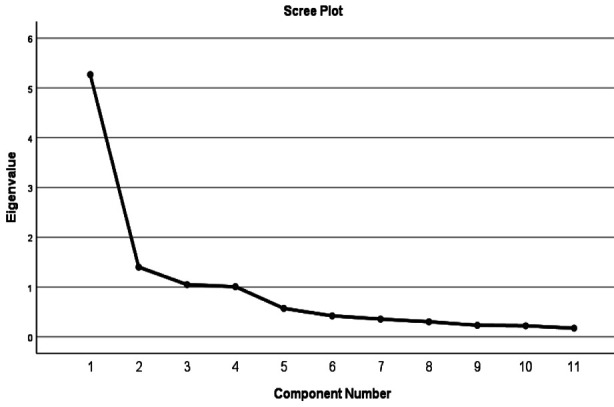
Scree Plot of Initial Analysis.

Details of correlation matrix of all questions of CLDQ Urdu are given in Table-II initial results of total variance identified four factors that were significant based on Eigenvalues of ≥1 these are detailed in Table-III and graphically in Fig.1.

**Table-II:** Correlation Matrix of CLDQ Urdu.

	Abdominal Bloating	Tired or Fatigue	Body Pain	Sleepy during day	Abdominal Pain	Shortness of breath	Unable to eat as desired	Decreased strength	Lifting heavy objects	Anxious	Decreases energy	Unhappy	Drowsy	Limitation of diet	Irritable	Difficulty to Sleep at night	Abdominal Discomfort	Impact of liver disease on family	Mood swings	Unable to sleep at night	Muscle cramps	Worry for development of major problem	Dry mouth	Depressed	Condition getting worse	Problem in concentrating	Itching	Never getting better	Availability of liver for transplant
Abdominal Bloating	1.0	.447	.419	.381	.446	.393	.256	.376	.383	.394	.364	.387	.315	.350	.363	.333	.481	.482	.289	.256	.341	.373	.209	.389	.383	.317	.284	.422	.245
Tired or Fatigue	.447	1.0	.714	.389	.363	.437	.405	.676	.514	.455	.665	.448	.329	.398	.410	.372	.415	.455	.311	.327	.496	.424	.299	.394	.471	.375	.234	.438	.126
Body Pain	.419	.714	1.0	.442	.385	.500	.409	.761	.556	.443	.713	.506	.425	.422	.433	.360	.380	.444	.355	.343	.544	.474	.382	.452	.446	.392	.209	.384	.176
Sleepy during day	.381	.389	.442	1.0	.292	.301	.314	.378	.343	.323	.354	.402	.679	.318	.347	.355	.232	.447	.289	.311	.277	.314	.255	.406	.388	.399	.262	.279	.092
Abdominal Pain	.446	.363	.385	.292	1.0	.448	.295	.409	.407	.268	.353	.298	.284	.326	.288	.291	.752	.431	.224	.236	.353	.370	.239	.307	.358	.271	.227	.357	.100
Shortness of breath	.393	.437	.500	.301	.448	1.0	.404	.490	.564	.467	.482	.482	.284	.380	.444	.423	.398	.384	.418	.406	.413	.441	.299	.446	.402	.428	.288	.358	.184
Unable to eat as desired	.256	.405	.409	.314	.295	.404	1.0	.339	.411	.377	.334	.312	.321	.306	.306	.297	.327	.329	.302	.291	.259	.339	.254	.246	.306	.386	.227	.280	.100
Decreased strength	.376	.676	.761	.378	.409	.490	.339	1.0	.515	.432	.806	.490	.386	.434	.405	.386	.383	.448	.342	.403	.510	.428	.372	.440	.460	.423	.162	.409	.148
Lifting heavy objects	.383	.514	.556	.343	.407	.564	.411	.515	1.0	.567	.536	.482	.372	.433	.481	.424	.409	.413	.453	.465	.391	.468	.414	.441	.451	.481	.249	.374	.188
Anxious	.394	.455	.443	.323	.268	.467	.377	.432	.567	1.0	.440	.612	.315	.417	.556	.517	.297	.453	.550	.524	.336	.494	.388	.554	.511	.457	.298	.459	.217
Decreases energy	.364	.665	.713	.354	.353	.482	.334	.806	.536	.440	1.0	.509	.356	.374	.428	.414	.343	.494	.366	.351	.497	.465	.374	.445	.417	.392	.134	.394	.119
Unhappy	.387	.448	.506	.402	.298	.482	.312	.490	.482	.612	.509	1.0	.469	.473	.647	.501	.290	.531	.622	.489	.294	.628	.490	.811	.564	.412	.285	.445	.237
Drowsy	.315	.329	.425	.679	.284	.284	.321	.386	.372	.315	.356	.469	1.0	.459	.343	.384	.219	.394	.309	.341	.229	.402	.293	.410	.430	.488	.297	.270	.111
Limitation of diet	.350	.398	.422	.318	.326	.380	.306	.434	.433	.417	.374	.473	.459	1.0	.413	.460	.299	.401	.358	.412	.284	.475	.320	.436	.424	.396	.268	.388	.214
Irritable	.363	.410	.433	.347	.288	.444	.306	.405	.481	.556	.428	.647	.343	.413	1.0	.545	.254	.474	.621	.439	.334	.517	.415	.626	.532	.406	.352	.371	.251
Difficulty to Sleep at night	.333	.372	.360	.355	.291	.423	.297	.386	.424	.517	.414	.501	.384	.460	.545	1.0	.280	.436	.483	.553	.265	.464	.313	.523	.458	.454	.238	.395	.190
Abdominal Discomfort	.481	.415	.380	.232	.752	.398	.327	.383	.409	.297	.343	.290	.219	.299	.254	.280	1.0	.408	.216	.227	.342	.425	.254	.291	.402	.262	.296	.341	.150
Impact of liver disease on family	.482	.455	.444	.447	.431	.384	.329	.448	.413	.453	.494	.531	.394	.401	.474	.436	.408	1.0	.439	.397	.306	.512	.393	.505	.551	.459	.271	.485	.225
Mood swings	.289	.311	.355	.289	.224	.418	.302	.342	.453	.550	.366	.622	.309	.358	.621	.483	.216	.439	1.0	.498	.168	.511	.397	.601	.514	.420	.277	.383	.233
Unable to sleep at night	.256	.327	.343	.311	.236	.406	.291	.403	.465	.524	.351	.489	.341	.412	.439	.553	.227	.397	.498	1.0	.243	.378	.405	.476	.463	.436	.230	.342	.155
Muscle cramps	.341	.496	.544	.277	.353	.413	.259	.510	.391	.336	.497	.294	.229	.284	.334	.265	.342	.306	.168	.243	1.0	.419	.322	.311	.439	.335	.202	.387	.039
Worry for development of major problem	.373	.424	.474	.314	.370	.441	.339	.428	.468	.494	.465	.628	.402	.475	.517	.464	.425	.512	.511	.378	.419	1.0	.480	.652	.700	.467	.340	.533	.182
Dry mouth	.209	.299	.382	.255	.239	.299	.254	.372	.414	.388	.374	.490	.293	.320	.415	.313	.254	.393	.397	.405	.322	.480	1.0	.489	.424	.321	.244	.352	.072
Depressed	.389	.394	.452	.406	.307	.446	.246	.440	.441	.554	.445	.811	.410	.436	.626	.523	.291	.505	.601	.476	.311	.652	.489	1.0	.590	.417	.298	.465	.200
Condition getting worse	.383	.471	.446	.388	.358	.402	.306	.460	.451	.511	.417	.564	.430	.424	.532	.458	.402	.551	.514	.463	.439	.700	.424	.590	1.0	.486	.355	.600	.192
Problem in concentrating	.317	.375	.392	.399	.271	.428	.386	.423	.481	.457	.392	.412	.488	.396	.406	.454	.262	.459	.420	.436	.335	.467	.321	.417	.486	1.0	.315	.438	.216
Itching	.284	.234	.209	.262	.227	.288	.227	.162	.249	.298	.134	.285	.297	.268	.352	.238	.296	.271	.277	.230	.202	.340	.244	.298	.355	.315	1.0	.255	.141
Never getting better	.422	.438	.384	.279	.357	.358	.280	.409	.374	.459	.394	.445	.270	.388	.371	.395	.341	.485	.383	.342	.387	.533	.352	.465	.600	.438	.255	1.0	.195
Availability of liver for transplant	.245	.126	.176	.092	.100	.184	.100	.148	.188	.217	.119	.237	.111	.214	.251	.190	.150	.225	.233	.155	.039	.182	.072	.200	.192	.216	.141	.195	1.0

**Table-III:** Initial Table of Total Variance.

Total Variance Explained									

Component	Initial Eigenvalues			Extraction Sums of Squared Loadings			Rotation Sums of Squared Loadings		
		
	Total	% of Variance	Cumulative %	Total	% of Variance	Cumulative %	Total	% of Variance	Cumulative %
1	5.267	47.878	47.878	5.267	47.878	47.878	2.791	25.376	25.376
2	1.401	12.737	60.615	1.401	12.737	60.615	2.414	21.947	47.324
3	1.048	9.532	70.147	1.048	9.532	70.147	1.766	16.053	63.377
4	1.007	9.153	79.300	1.007	9.153	79.300	1.752	15.923	79.300
5	.571	5.188	84.488						
6	.421	3.830	88.318						
7	.356	3.237	91.555						
8	.302	2.743	94.298						
9	.232	2.105	96.404						
10	.221	2.013	98.417						
11	.174	1.583	100.000						

Extraction Method: Principal Component Analysis.

Initial Rotated Component Matrix showed many communalities having loadings on multiple factors. All items with cross loadings were excluded, and analysis was run again. The results obtained are shown in Table-IV.

**Table-IV:** Results of Exploratory Factor Analysis after factor reduction

Rotated Component Matrix^a^					

	Items	Component			

		1	2	3	4
1.	Body Pain	.811			
2.	Tired or Fatigue	.793			
3.	Decreased strength	.793			
4.	Muscle cramps	.727			
5.	Depressed		.859		
6.	Unhappy		.850		
7.	Irritable		.791		
8.	Abdominal Discomfort			.893	
9.	Abdominal Pain			.888	
10.	Drowsy				.864
11.	Sleepy during day				.856

Extraction Method: Principal Component Analysis. Rotation Method: Varimax with Kaiser Normalization. a. Rotation converged in 5 iterations.

Exploratory factor analysis after factor reduction identified four main components, which were named on basis of factors included, ***‘Muscular Symptoms’*** which includes Body Pain, Tired or Fatigue, Decreased Strength, and Muscle Cramps. The second component was named ***‘Emotional Symptoms’*** which included Depressed, Unhappy & Irritable. Third component was named ***‘Abdominal Symptoms’***, and it included Abdominal Discomfort & Abdominal Pain. Fourth component was named ***‘Somnolence’*** it included Drowsy & Sleepy during day. Their values are given in Table-IV. The percentage of variance for weakness and pain was 47.88, depression was 12.74, abdominal pain was 9.53 and for somnolence was 9.15 and their Eigen values were 5.27, 1.40,1.05 & 1.01 respectively. The cumulative variance explained by these four components was 79.30%.

Mean CLDQ Urdu score was 156.74 in male and 133.27 in female patients showing better perception of health in males as compared to female (*p*<.001). Mean CLDQ Urdu scores according to Child Class are given in Table-V. The combined differences between Child Class were assessed by ANOVA test and it showed that the differences between groups was significantly different (p < .001). Child Class A had best quality of life with score of 186.63 ±6.91 and Child Class C having the worst with scores of 109.78 ±21.33.

**Table-V:** Mean CLDQ Urdu scores according to Child Class.

	N	Mean	SD	95% Confidence Interval for Mean	

				Lower Bound	Upper Bound
Child Class A	86	186.63	6.91	185.15	188.11
Child Class B	113	159.56	10.48	157.60	161.51
Child Class C	121	109.78	21.33	105.94	113.62

Total	320	148.01	35.02	144.16	151.86

There was significant progressive decrease in CLDQ Urdu scores from Child Class A to C showing decrease in QOL. The scores decreased from Child Class A to B by 27.07; from B to C by 49.78 all were statistically significant (p <.001). Details are in Table-VI.

**Table-VI:** Post Hoc ANOVA analysis of CLDQ Urdu scores between Child Class Groups.

(I) Child Class	(J) Child Class	Mean Difference (I-J)	Sig.	95% Confidence Interval	

				Lower Bound	Upper Bound
Child Class A	Child Class B	27.07*	<.001	24.152	29.989
	Child Class C	76.85*	<.001	71.935	81.768
Child Class B	Child Class A	-27.07*	<.001	-29.989	-24.152
	Child Class C	49.78*	<.001	44.638	54.923
Child Class C	Child Class A	-76.85*	<.001	-81.768	-71.935
	Child Class B	-49.78*	<.001	-54.923	-44.638

* Mean difference is significant at 0.05 level.

### Limitations:

The exploratory factor analysis of our Urdu version of CLDQ showed high internal validity and consistency and reduced factor items from 29 to 4 which explained a79.3 % of total variance. This being single center study need to be validated in multi center design.

## DISCUSSION

Chronic liver disease carries a huge burden at health care in Pakistan leading to many complications like ascites, encephalopathy, and varices.^14^-^16^ Assessment of quality of life in these patients is of outmost importance. There are many modalities and different ways for treatment of physical ailments of a patient but that is not all needed for complete recovery of patient from any illness. Instead of only physical health, global assessment of heath is necessary including psychological, social and mental health. Tools have been developed by which severity and progression and quality of life of CLD patients can be assessed but unfortunately none is available in our national language, i.e., Urdu. HRQOL has been assessed and reported in many diseases like dental, ischemic vascular, liver and neurological diseases in Pakistan but they were all done in English language and were done with the assistance of the investigator.^10^,^17^-^19^ Up till now none was done with native language spoken in Pakistan.

Our Urdu CLDQ version is easier, simple, reliable, and excellent way for the assessment of health-related quality of life of CLD patients as demonstrated in current study. This study demonstrates good reliability and statically significant internal consistency which is compatible with Original CLDQ. It is evident from our study that Urdu CLDQ version is theoretically and conceptually equivalent to the original one. It not only provides overall well-being of CLD patient but also helps in assessment of disease progression and effect of management on patient health with minimum items. Though the validity of CLDQ has been confirmed by many studies but exploratory factor analysis was done in Spanish,^11^ Greek^20^ and Italian^12^ studies only. There is remarkable change in the structure of CLDQ after exploratory factor analysis in our study. We are left with only four main domains instead of 6 in original and Italian^12^ version and seven domains in Spanish^11^ and Greek^20^ versions of CLDQ. The new domains are labelled based on factors incorporated into them as ***Muscular Symptoms, Emotional Symptoms, Abdominal Symptoms & Somnolence***. Factor analysis not only reduced the number of domain but also compacted the number of items from 29 to 11.

CLQ URDU QUESTIONNAIRE AFTER FACTOR REDUCTION


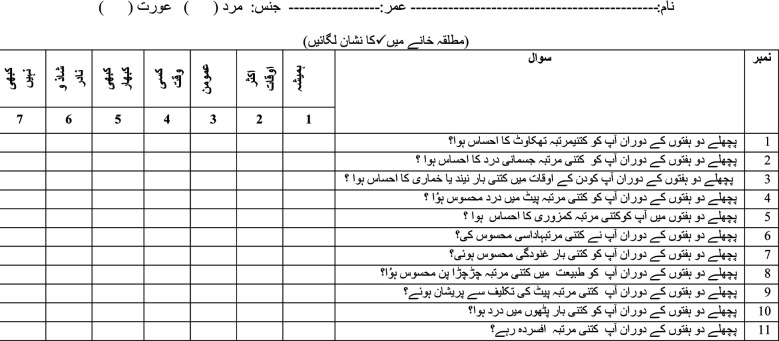


Factors included in *‘Muscular Symptoms’* were Body Pain, Tired or Fatigue, Decreased Strength & Muscle Cramps, these were included in Fatigue and Systemic Symptoms in original version.^3^ Our second domain is *‘Emotional Symptoms’* and it included Depressed, Unhappy and Irritable all three were also under the domain of Emotional Symptoms in Original,^3^ Spanish^11^ and Greek versions.^20^ Our third domain is *‘Abdominal Symptoms’* and it includes abdominal discomfort and abdominal pain, these were also included in Abdominal Symptoms domain in original,^3^ Spanish^11^ and Greek^20^ questionnaire. Our last domain is *‘Somnolence’* it included Drowsy & Sleepy during day, it was included in Fatigue in Original CLDQ.

This type of study has never been conducted in Pakistan. Many versions of CLDQ have been published in different languages of the world but this is first time that CLDQ is translated in Urdu language which help us in evaluation of health-related quality of life of CLD patient in much better way in our population and that is the main and the major strength of this study. Contrary to some studies in which CLDQ is applied only to of CLD patients of selected etiologies,^21^-^24^ our study has included all the patient of CLD so is not limited to certain group of CLD patient. Urdu CLDQ version enables us to effectively assess the quality of CLD patient even without applying any scoring system.

### Limitations:

One of the limitations of current study was its being a single center study. As we have excluded patients with hepatic encephalopathy and HCC from the study, we are not able to comment on health-related quality of life of patient with hepatic encephalopathy and HCC. Data of CLD patients who had undergone liver transplantation or waiting for transplantation is also lacking because of non-availability of transplantation facility at place where study is conducted.

## CONCLUSION

Urdu CLDQ version is a practical and effective tool for evaluation of health-related quality of CLD patients. It is recommended that Urdu CLDQ version be included in routine assessment of all CLD patient as a general predictor of their quality of their health and response to treatment. Validity of Urdu CLDQ version is established and confirmed in our study. Exploratory factor analysis resulted in a simplified version of original CLDQ version with only eleven items. This will ease the assessment of quality of life in patients with chronic liver disease

### Author`s Contribution

**SMH:** Data Collection, responsible for integrity of data and study. **BFZ:** Statistical analysis, final approval and editing of manuscript. **TR:** Conception of study, data analysis. **FSA:** Initial draft of manuscript. **EM:** Data collection and manuscript editing
